# Diagnostic Yield and Clinical Phenotype in a Lithuanian Familial Hypercholesterolemia Cohort with Heterogeneous Sequencing Platforms

**DOI:** 10.3390/medicina62101891

**Published:** 2026-09-29

**Authors:** Kristina Zubielienė, Darius Čereskevičius, Diana Žaliaduonytė, Vacis Tatarūnas, Neda Jonaitienė, Marius Šukys, Ingrida Grabauskytė, Tautvydas Kabošis, Rasa Ugenskienė, Vaiva Lesauskaitė, Rimvydas Šlapikas, Ugnė Meškauskaitė, Monika Biesevičienė, Ieva Aleknaitė, Gabrielė Žūkaitė, Gabrielė Žebrauskaitė-Keblikienė, Vytautas Zabiela

**Affiliations:** 1 Heart Center, Kaunas Hospital, Lithuanian University of Health Sciences, LT-50161 Kaunas, Lithuania; 2 Heart Center, Hospital of Lithuanian University, Health Sciences Kauno Klinikos, LT-50161 Kaunas, Lithuania; 3 Kaunas Region Society of Cardiology, LT-50161 Kaunas, Lithuania; 4 Institute of Cardiology, Lithuanian University of Health Sciences, LT-50161 Kaunas, Lithuania; 5 Department of Genetics and Molecular Medicine, Lithuanian University of Health Sciences, LT-50161 Kaunas, Lithuania; 6 Department of Physics, Mathematics and Biophysics, Lithuanian University of Health Sciences, LT-50161 Kaunas, Lithuania; 7 Faculty of Medicine, Lithuanian University of Health Sciences, LT-50161 Kaunas, Lithuania

**Keywords:** familial hypercholesterolemia, genetic testing, pharmacogenomics, statins

## Abstract

*Background and Objectives:* This study aimed to determine the diagnostic yield of familial hypercholesterolemia (FH) genotyping and to describe the clinical phenotype and pharmacogenomic (PGx) implementation readiness of adults referred for clinically suspected FH at a Lithuanian tertiary cardiology center. The study was designed as a descriptive retrospective analysis of existing clinical and genetic test results generated using heterogeneous historical sequencing platforms. *Materials and Methods:* This single-center descriptive cohort included 48 adults with clinically suspected FH evaluated between February 2022 and June 2023. Patients were classified according to Dutch Lipid Clinic Network criteria before genetic testing. Historical targeted next-generation sequencing (NGS) and whole-exome sequencing (WES) results were reviewed. Genetic findings were interpreted using a three-tier reporting framework: diagnostic FH-associated findings, pharmacogenomic findings relevant to future implementation, and VUS, common polymorphisms, or other non-diagnostic findings. Because harmonized PGx star-allele/diplotype calling was not available across historical platforms, PGx findings were not analysed as a mutually exclusive patient-level category. *Results:* Pathogenic FH-associated variants were identified in 13 of 48 patients (27.1%), including eight *LDLR* and five *APOB* variant carriers. In the remaining 35 patients (72.9%), no pathogenic or likely pathogenic FH-associated variant was identified within the analytical scope of the historical test used. This non-diagnostic category could include VUS, common polymorphisms, exploratory findings, or no reportable variant. Patients carrying pathogenic *LDLR* variants had higher total cholesterol and LDL-C concentrations than patients without detected pathogenic variants. PGx-related findings were considered observations relevant to reporting and future implementation; they were not evaluated as predictors of statin response, statin intolerance, or LDL-C reduction. *Conclusions:* In this Lithuanian real-world cohort, pathogenic *LDLR* or *APOB* variants were identified in 27.1% of patients referred for suspected FH. Retrospective analyses of heterogeneous historical genetic results can inform diagnostic reporting and implementation planning but cannot establish PGx associations with statin efficacy or intolerance without harmonized diplotype calling and prospectively collected treatment outcomes.

## 1. Introduction

Familial hypercholesterolemia (FH) is an inherited disorder of lipoprotein metabolism characterized by lifelong elevation of low-density lipoprotein cholesterol (LDL-C) and increased risk of premature atherosclerotic cardiovascular disease [1,2]. Early recognition of FH has direct clinical value because it enables timely lipid-lowering therapy, identification of an index case and cascade testing of relatives [1,2,3]. In precision lipidology, genetic testing is therefore not only a diagnostic tool but also an entry point into individualized prevention [2,3].

Most monogenic FH is caused by pathogenic variants in *LDLR,* whereas pathogenic *APOB* variants and gain-of-function *PCSK9* variants are less frequent [1,2,3,4,5]. In patients with a clinical FH phenotype but no detected pathogenic variant, possible explanations include polygenic hypercholesterolemia, undetected structural variants, other lipid disorders, secondary causes or a combination of conventional cardiovascular risk factors [3,4,6]. Clinical criteria such as the Dutch Lipid Clinic Network (DLCN) score remain important because a negative sequencing result does not exclude clinically relevant hypercholesterolemia [7].

The pharmacogenomic dimension is increasingly relevant in FH because patients usually require lifelong and intensive lipid-lowering treatment [8,9,10,11,12]. Statins remain foundational therapy, and pharmacogenomic variation in genes such as SLCO1B1, ABCG2 and CYP2C9 can influence statin exposure and the risk of statin-associated musculoskeletal symptoms [8]. The clinical aim of statin pharmacogenomics is not to avoid indicated therapy, but to support safer statin selection, dose adjustment, monitoring and adherence when actionable genotype results are available [8,13].

In small real-world cohorts, the challenge is not only technical sequencing but also interpretation [14,15,16,17,18]. A single NGS workflow may generate diagnostic variants, variants of uncertain significance (VUS), common polymorphisms and exploratory pharmacogenomic findings [14,15,16]. Treating all these results as equivalent can mislead clinicians [14]. Conversely, excluding pharmacogenomic information entirely may miss opportunities for individualized prescribing [8,15,16]. This tension is particularly relevant in countries or centers where pharmacogenomic implementation is still developing [15,16,17,18].

This manuscript reframes a Lithuanian suspected FH cohort as a real-world implementation case study. Rather than attempting to identify independent acute coronary syndrome (ACS) predictors in this descriptive cohort based on retrospectively analyzed records, we focus on what such a cohort can validly show: diagnostic yield, clinical phenotype, pharmacogenomic readiness, and the safeguards required before NGS-derived information can be used for precision medicine [15,16,17,18].

Therefore, the aim of this study was to quantify the diagnostic yield of FH genotyping and to describe the clinical phenotype and pharmacogenomic implementation readiness in adults referred for clinically suspected FH at a Lithuanian tertiary cardiology center. In this manuscript, “small real-world cohorts” refers to a single-center descriptive cohort of referred FH patients evaluated in routine clinical practice.

## 2. Materials and Methods

### 2.1. Study Design and Population

This was a single-center descriptive cohort study of adults referred for clinically suspected familial hypercholesterolemia (FH) to the Department of Cardiology of the Lithuanian University of Health Sciences between February 2022 and June 2023. The present analysis was based on retrospectively reviewed existing clinical records, lipid measurements, and genetic test results obtained during routine clinical evaluation. Patients evaluated before ethics approval on 11 August 2022 were identified retrospectively through chart review and existing clinical and lipid data. Although a subset of patients was enrolled prospectively during routine care after ethics approval, the present study is a retrospective analysis of existing clinical, lipid, and genetic test records. No prospective protocol was used to collect standardized treatment-response or pharmacogenomic outcome data for the analyses reported here. Genetic test results had been generated during routine clinical evaluation using historical, non-uniform sequencing workflows, including targeted next-generation sequencing (NGS) panels and whole-exome sequencing (WES). The study was not designed to compare the diagnostic performance of the two platforms. Accordingly, negative or non-diagnostic findings were interpreted cautiously, taking into account potential differences in gene content, exon-level coverage, reference genome build, and analytical scope between historical testing approaches.

**Figure 1:**
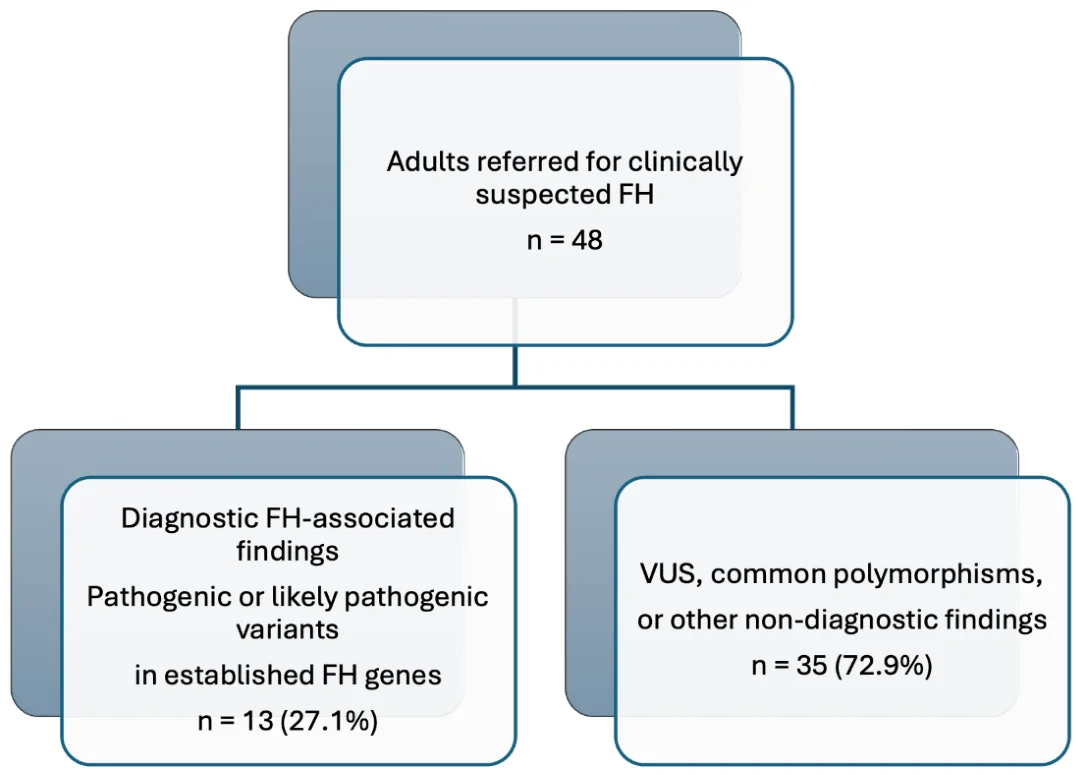
Genetic reporting categories in adults referred for clinically suspected familial hypercholesterolemia. Pathogenic or likely pathogenic variants in established FH genes were classified as diagnostic FH-associated findings. All remaining results were classified as VUS, common polymorphisms, or other non-diagnostic findings. FH—familial hypercholesterolemia, VUS—variants of uncertain significance.

**Figure 2:**
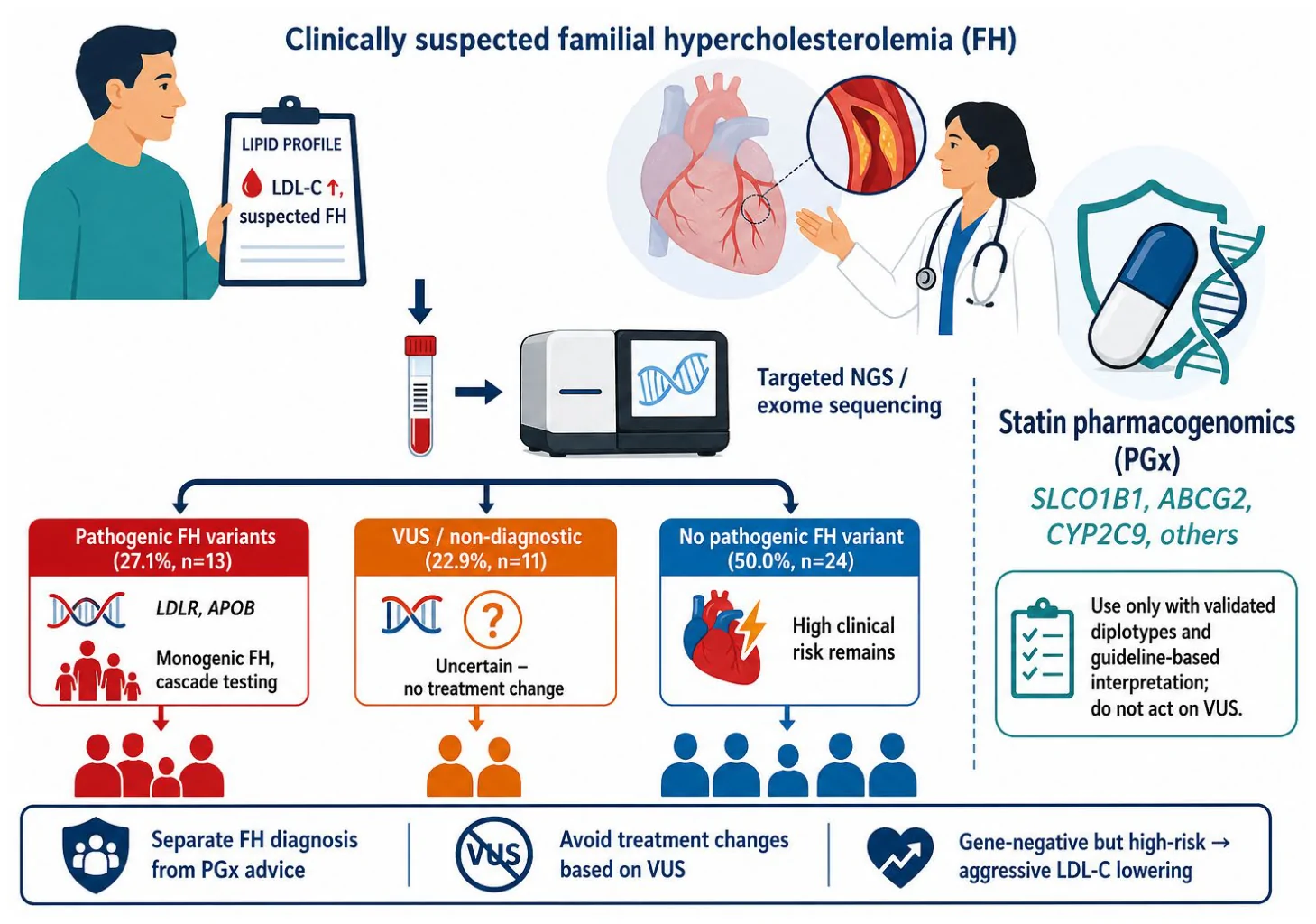
Overview of historical sequencing workflows used for adults referred for clinically suspected familial hypercholesterolemia, including targeted NGS panels and whole-exome sequencing.

Figure 1 summarizes the overall study flow, and Figure 2 provides a detailed overview of the sequencing workflow for adults referred for clinically suspected FH. The final cohort consisted of 48 patients.

Inclusion criteria were age 18–65 years, clinical suspicion of FH, and untreated or qualifying low-density lipoprotein cholesterol (LDL-C) ≥ 4.9 mmol/L. Exclusion criteria were pregnancy or breastfeeding, end-stage oncological or somatic disease, secondary conditions that may mimic the FH phenotype, such as hypothyroidism, nephrotic syndrome, or cholestasis, and absence of signed informed consent for participation and genetic testing. The study protocol was approved by the Kaunas Regional Biomedical Research Ethics Committee (Protocol No. BE-2-47; approval date: 11 August 2022).

### 2.2. Clinical and Lipid Assessment

Medical records were reviewed for age, sex, body mass index (BMI), smoking status, arterial hypertension, diabetes mellitus, lipid profile, lipid-lowering therapy status, personal history of ischemic heart disease, family history and ACS status. Lipid variables included total cholesterol (TC), LDL-C, high-density lipoprotein cholesterol (HDL-C) and triglycerides (TG).

ACS status was determined based on the index cardiology evaluation. In patients with ACS, lipid values were obtained during the index hospitalization for the acute coronary event, before initiation of lipid-lowering therapy. In patients without ACS, the highest available untreated LDL-C value before lipid-lowering therapy was used for FH suspicion and DLCN assessment. Because acute-phase lipid values are not necessarily equivalent to untreated baseline lipid exposure, lipid findings were interpreted descriptively and cautiously [19,20].

### 2.3. Clinical FH Classification

Before genetic testing, patients were classified according to the DLCN criteria. DLCN categories were interpreted as possible FH for 3–5 points, probable FH for 6–8 points and definite FH for more than 8 points [7]. Genetic testing results were subsequently interpreted separately from the pre-genetic clinical score.

### 2.4. Genetic and Pharmacogenomic Testing Workflow

Peripheral venous blood was collected in EDTA tubes. Genomic DNA was extracted by salting-out for targeted-panel samples or using the QIAsymphony DNA Kit on a QIAsymphony SP instrument (QIAGEN, Hilden, Germany) for whole-exome sequencing (WES) samples. Targeted sequencing was performed in-house in 36 patients (75.0%). Libraries were prepared using the Illumina DNA Prep with Enrichment kit (Illumina, San Diego, CA, USA) and a custom xGen Lockdown hybridisation capture panel (Integrated DNA Technologies, Coralville, IA, USA) targeting canonical coding exons and flanking splice regions. The panel included FH-associated genes (*LDLR*, *APOB*, *PCSK9*, and *LDLRAP1*) and genes involved in statin pharmacokinetics and pharmacodynamics. Libraries were sequenced on an Illumina MiniSeq platform (Illumina, San Diego, CA, USA) using 2 × 150 bp paired-end reads. All coding bases were covered at ≥20× in every sample. Reads were aligned to GRCh38 using BWA-MEM (version 0.7.17) and processed with the Genome Analysis Toolkit (GATK; version 4.5.0.0; Broad Institute, Cambridge, MA, USA) according to GATK Best Practices. Single-nucleotide variants and small insertions/deletions were called using GATK HaplotypeCaller and filtered using GATK hard-filtering thresholds. Copy-number variants (CNVs) were assessed with CNVkit (version 0.9.9) using a pooled reference from 30 independently sequenced samples with the same panel, library chemistry, and instrument [21,22].

WES was performed in 12 patients (25.0%) by CeGaT GmbH (Tübingen, Germany). Libraries were prepared with Twist Bioscience kits (South San Francisco, CA, USA), enriched using the provider’s exome panel, and sequenced on an Illumina NovaSeq platform (Illumina, San Diego, CA, USA) with 2 × 100 bp paired-end reads to a mean on-target coverage of 150×. Variant and read-depth-based CNV calling were performed using the Illumina DRAGEN Bio-IT Platform (version 4.1 or later). Depending on the provider’s reference build at the time of analysis, samples were aligned to either GRCh37/hg19 or GRCh38. Variants were reported using HGVS nomenclature based on the reference transcripts specified below, ensuring consistency across genome builds. Analysis was restricted to a virtual panel containing the same genes as the targeted panel. CNV analysis was performed in all patients using CNVkit for targeted-panel data and the DRAGEN read-depth caller for WES data. Exon-level deletions and duplications, including those affecting *LDLR*, were within the scope of both workflows. No pathogenic CNVs were identified in *LDLR*, *APOB*, *PCSK9*, or *LDLRAP1*. CNV calls were not confirmed using an orthogonal method, such as multiplex ligation-dependent probe amplification. Variants passing site-level filters were annotated and prioritised using Franklin by Genoox (Genoox, Palo Alto, CA, USA; https://franklin.genoox.com, accessed on 30 June 2026), based on the reference transcripts NM_000527.5 (*LDLR*), NM_000384.3 (*APOB*), NM_174936.4 (*PCSK9*), and NM_015627.3 (*LDLRAP1*). Prioritisation considered population allele frequency, predicted consequence, and position within coding or canonical splice regions. No allele-frequency filter was applied to pharmacogenetic loci because clinically relevant alleles may be common in the general population. Synonymous variants without predicted splice effects were excluded. Automated classifications were manually reviewed and finalised according to ACMG/AMP guidelines, applying ClinGen Familial Hypercholesterolemia Variant Curation Expert Panel specifications for *LDLR* where appropriate, with support from ClinVar (https://www.ncbi.nlm.nih.gov/clinvar/, accessed on 30 June 2026). Variants were not confirmed by Sanger sequencing. Only pathogenic or likely pathogenic variants in *LDLR*, *APOB*, *PCSK9*, or *LDLRAP1* were considered diagnostic FH-associated findings. Because VUS reporting differed between the historical platforms and complete original variant-level reports were not uniformly available for all patients, the number of patients carrying at least one VUS could not be reconstructed reliably across the full cohort. Therefore, VUS were retained within the composite category of VUS, common polymorphisms, or other non-diagnostic findings. Pharmacogenetic findings were reported as individual variants. Star-allele/diplotype calling was not performed, and short-read sequencing does not reliably resolve the structurally complex *CYP2D6* locus. Therefore, pharmacogenetic findings were interpreted as implementation-readiness observations rather than validated predictors of drug response. A summary of the two historical sequencing workflows, including the number of patients tested with each platform, analytical scope, coverage information, reference genome, and CNV analysis, is provided in Supplementary Table S1.

### 2.5. Coronary Angiography

Coronary angiography was performed when clinically indicated in patients with ACS. Affected coronary segments were summarized descriptively. Because segment-level observations are clustered within patients and the cohort was small, angiographic findings were not used for inferential genotype-specific modelling.

### 2.6. Statistical Analysis

Statistical analyses were performed using IBM SPSS Statistics, version 32.0.0 (IBM Corp. Armonk, NY, USA) and were descriptive by design. The distribution of continuous variables was assessed using the Shapiro–Wilk test. Continuous variables are presented as mean (range) when normally distributed and as medians with interquartile ranges (IQR) when non-normally distributed. Categorical variables are presented as counts and percentages. Group comparisons for continuous variables were performed using the Mann–Whitney U test for two-group comparisons and the Kruskal–Wallis test for comparisons across more than two groups, with Dunn’s post hoc test when appropriate. Categorical variables were compared using the chi-square test or Fisher’s exact test, depending on expected cell counts. A two-sided *p*-value < 0.05 was considered statistically significant. To avoid unstable estimates and misleading inference in this small, selected cohort, no multivariable regression modelling was performed; instead, lipid and genetic findings were interpreted descriptively in the clinical and implementation context. Formal a priori statistical power calculation was not performed because the cohort size was determined by the available referred patients during the study period and the study was framed as a descriptive implementation analysis rather than a hypothesis-testing trial.

## 3. Results

### 3.1. Cohort Characteristics and ACS Status

The cohort included 48 adults referred for clinically suspected FH. ACS was present in 28 patients (58.3%) and absent in 20 patients (41.7%). Among patients with ACS, 18 were male and 10 were female. Patients with ACS were older and more frequently had arterial hypertension. TC and LDL-C values were lower in the ACS group, whereas TG values were higher. Because lipid values in patients with ACS were obtained during the index acute event, whereas qualifying values in patients without ACS were based on the highest available untreated LDL-C measurement before lipid-lowering therapy, these between-group lipid comparisons are descriptive and should not be interpreted as reflecting differences in lifetime lipid exposure or treatment-independent genetic effects. Baseline clinical characteristics according to ACS status are summarized in Table 1.

### 3.2. Diagnostic FH Genetic Findings

Of the 48 patients, 36 (75.0%) were analysed with the targeted NGS panel and 12 (25.0%) with whole-exome sequencing; the analytical characteristics and limitations of the historical workflows are summarized in Supplementary Table S1. Both workflows were designed to assess the canonical coding exons and flanking canonical splice regions of *LDLR*, *APOB*, *PCSK9*, and *LDLRAP1* and included read-depth-based CNV analysis. In the targeted-panel workflow, all intended coding bases were covered at ≥20×. For WES, the laboratory-reported mean on-target coverage was 150×; however, harmonized gene- and exon-level coverage metrics were not retrospectively available for all WES samples. Pathogenic or likely pathogenic FH-associated variants were identified in 13 of 48 patients (27.1%). Diagnostic and non-diagnostic genetic findings are summarized in Table 2.

*LDLR* variants were identified in eight patients: c.1013G>A (p.Cys338Tyr) in five patients, c.1829C>T (p.Ser610Phe) in two patients, and c.941-1_946del in one patient. A pathogenic *APOB* variant, c.10580G>A (p.Arg3527Gln), was identified in five patients. No pathogenic *PCSK9* variant was detected in this cohort.

In the remaining 35 patients (72.9%), no pathogenic or likely pathogenic FH-associated variant was identified within the analytical scope of the historical test used. This non-diagnostic group may include patients with VUS, common polymorphisms, other exploratory findings, or no reportable variant. Because VUS reporting and availability of detailed variant-level historical reports differed between testing platforms, the exact number of VUS carriers could not be reconstructed reliably for the entire cohort. The three-tier framework was applied at the level of result interpretation. However, because PGx star-allele/diplotype calling was not harmonized across the historical sequencing platforms, PGx findings were not treated as a mutually exclusive patient-level reporting category and were not included in the diagnostic versus non-diagnostic patient-level classification.

Patients carrying pathogenic *LDLR* variants had higher total cholesterol (10.37 mmol/L; *p* = 0.04) and LDL-C concentrations (7.91 mmol/L; *p* < 0.001) than patients without a detected pathogenic FH-associated variant. These findings suggest a more severe lipid phenotype among carriers of pathogenic *LDLR* variants in this cohort. Combining DLCN-based clinical assessment with genetic testing may therefore help identify individuals with a more severe monogenic FH phenotype.

Conversely, 35 of 48 patients (72.9%) had no identified pathogenic or likely pathogenic FH-associated variant within the analytical scope of the historical test used, underscoring that a substantial proportion of clinically suspected FH cases may reflect polygenic hypercholesterolemia, other genetic mechanisms not captured by the available testing approach, or multifactorial dyslipidemia.

### 3.3. Pharmacogenomic Readiness of the Sequencing Workflow

The broader sequencing workflow included genes that can be relevant to lipid-lowering pharmacotherapy or drug disposition, including transporter genes, metabolizing enzymes and candidate pharmacodynamic genes. Observed non-diagnostic findings included variants in ABCB1, UGT1A4, CYP2D6, UGT1A10 and HMGCR. A CYP3A5 c.-253-1A>G variant was detected in all individuals and was excluded from further FH interpretation because of its likely polymorphic nature. These findings illustrate a practical problem in PGx implementation: technically detected variants are not automatically clinically actionable. The pharmacogenomic interpretation framework for the sequencing workflow is presented in Table 3.

In the present cohort, however, PGx findings were not reported as harmonized star-allele diplotypes across all historical platforms, and no prospective statin-response or statin-intolerance outcomes were available. Therefore, the study should be interpreted as a readiness and reporting-framework analysis, not as a statin pharmacogenomic association study.

### 3.4. ACS Frequency by Genetic Category

**Figure 3:**
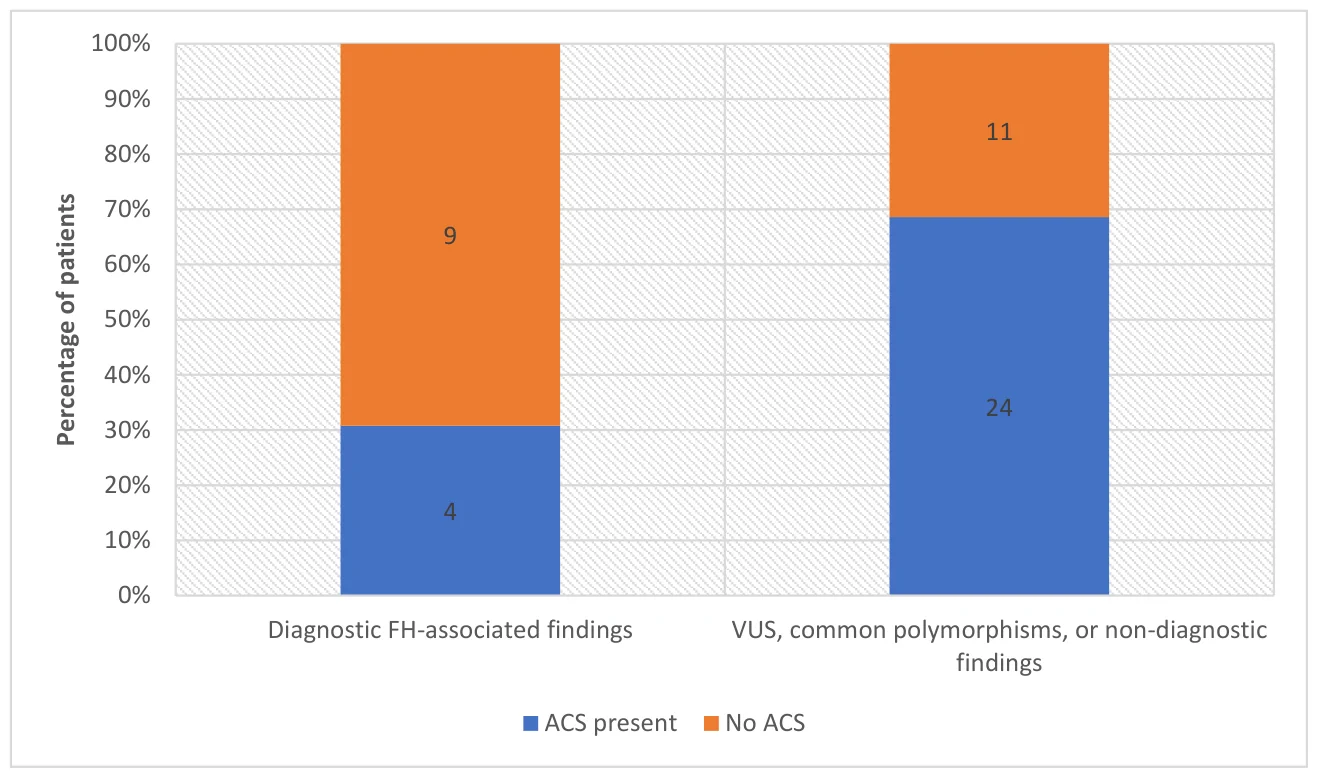
ACS status according to genetic reporting category. Percentages were calculated within each genetic reporting category. ACS—acute coronary syndrome; FH—familial hypercholesterolemia; VUS—variants of uncertain significance.

ACS was present in 4 of 13 patients (30.8%) with diagnostic FH-associated findings and in 24 of 35 patients (68.6%) with VUS, common polymorphisms, or other non-diagnostic findings. ACS frequencies according to genetic reporting category are presented in Figure 3.

These proportions are descriptive and should not be interpreted as genotype-specific risk estimates or as evidence that non-diagnostic findings are associated with a higher risk of ACS. The cohort was small and clinically selected, and data were analyzed retrospectively. In addition, lipid values in patients with ACS were obtained during the acute phase and may not reflect untreated baseline LDL-C exposure.

## 4. Discussion

**Figure 4:**
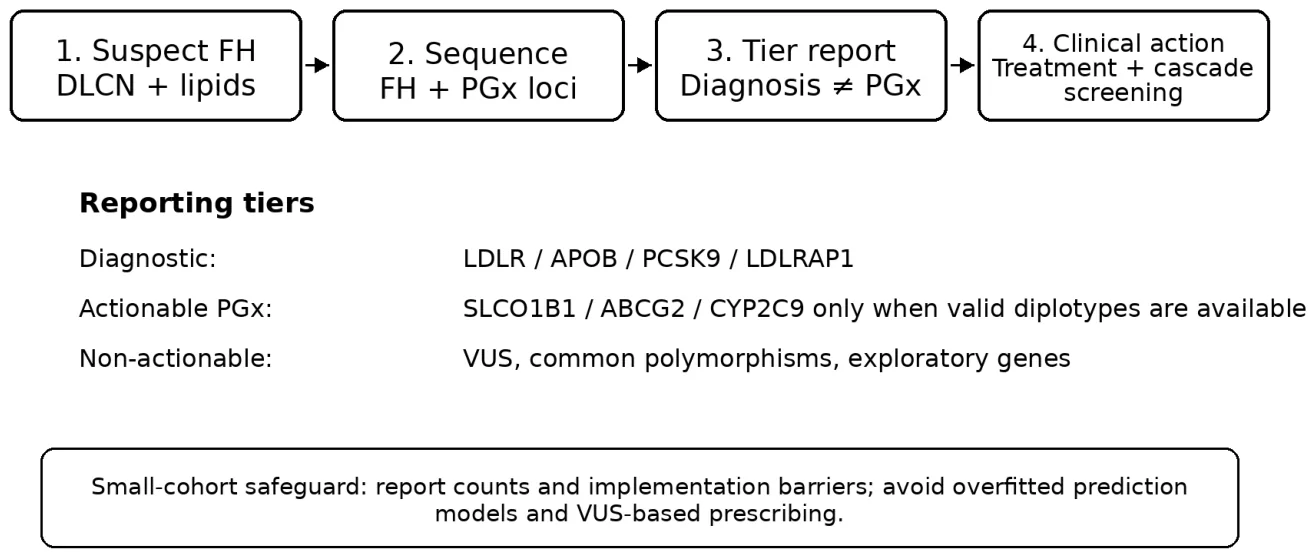
Proposed precision-medicine workflow separating FH diagnostic genetics from pharmacogenomic prescribing support. *ABCG2*—ATP-Binding Cassette subfamily G member 2, *APOB*—apolipoprotein B, *CYP2C9*—Cytochrome P450 family 2 subfamily C member 9, DLCN—Dutch Lipid Clinic Network, *PCSK9*—proprotein convertase subtilisin/kexin type 9, PGx—pharmacogenomic, *LDLR*—low-density lipoprotein receptor, *LDLRAP1*—Low-Density Lipoprotein Receptor Adaptor Protein 1, *SLCO1B1*—Solute Carrier Organic Anion Transporter family member 1B1, VUS—variants of uncertain significance.

This small, single-center descriptive cohort, based on retrospectively analyzed clinical and genetic records, illustrates how real-world FH data can inform pharmacogenomic implementation without overstating statistical inference. The diagnostic finding is straightforward: approximately one quarter of patients referred for suspected FH had a pathogenic variant in *LDLR* or *APOB*. This supports the value of targeted genetic testing in carefully selected patients and provides information that can be used for cascade screening. The pharmacogenomic message is more nuanced: the sequencing workflow can generate PGx-relevant information, but clinical use requires validated genotype calling, gene–drug guidance and appropriate reporting boundaries. A proposed precision-medicine workflow separating FH diagnostics from pharmacogenomic prescribing support is depicted in Figure 4.

The diagnostic yield observed in our cohort (27.1%) is broadly comparable with recent findings from neighboring Latvia. In a Latvian FH registry cohort assessed using genome-wide sequencing, pathogenic or likely pathogenic variants were identified in 20.9% of patients despite a high clinical likelihood of FH. A later combined monogenic and polygenic analysis reported causative variants in 23.6% of tested individuals and showed that polygenic scores explained an additional proportion of hypercholesterolemia among patients without monogenic findings [23,24]. These regional data support the interpretation that a substantial gene-negative subgroup is expected even in carefully selected Baltic FH cohorts and that negative results require cautious clinical interpretation rather than discontinuation of intensive lipid-lowering management.

More broadly, the diagnostic yield of FH genetic testing varies substantially across published cohorts because of differences in referral pathways, age, clinical diagnostic criteria, untreated LDL-C thresholds, family history, and the breadth of genetic testing. Cohorts enriched for patients with definite clinical FH or cascade-screened relatives generally report higher monogenic detection rates than unselected dyslipidaemia populations, whereas a substantial proportion of clinically suspected FH cases remain without an identifiable pathogenic variant. Therefore, the 27.1% diagnostic yield observed in the present cohort should be interpreted in relation to the selected tertiary-care referral setting and the historical heterogeneity of the testing workflows rather than as a population prevalence estimate [3,4,23,24,25].

The interpretation of non-diagnostic genetic results requires particular caution in this cohort. In the 35 patients without an identified pathogenic or likely pathogenic FH-associated variant, the result should not be interpreted as exclusion of a genetic contribution to hypercholesterolemia. Although the principal coding regions of the established FH genes and read-depth-based CNV analysis were within the analytical scope of both workflows, residual platform-specific limitations cannot be excluded, particularly for exon-level coverage variation, single-exon CNVs, deep intronic variants, regulatory variants, and findings outside the targeted analytical scope. Remaining explanations for a non-diagnostic result include polygenic hypercholesterolemia, variants in genes outside the analyzed panel, deep intronic or regulatory variants beyond the analytical scope of the targeted design, FH phenocopies, and multifactorial dyslipidemia [25]. Accordingly, VUS, common polymorphisms, and other non-diagnostic findings should not be used to diagnose monogenic FH or to guide cascade testing [26]. Nevertheless, patients with a clinically significant FH phenotype and no diagnostic variant still require phenotype- and risk-based management, including guideline-directed lipid-lowering therapy and control of other cardiovascular risk factors [1].

These considerations reinforce the need to distinguish diagnostic FH-associated findings from pharmacogenomic information and other non-diagnostic findings in the clinical report. *LDLR* and *APOB* pathogenic variants can confirm monogenic FH and guide family screening [2,3,4,5]. By contrast, variants in drug transporters or metabolizing enzymes may inform treatment only when the specific genotype is technically valid and clinically actionable for a particular medication [8,15,16]. The observation that many clinically suspected FH patients are ‘gene-negative’ highlights the continued importance of clinical FH criteria and aggressive LDL-C lowering based on overall cardiovascular risk, even in the absence of a detectable monogenic variant. Clinically, this gene-negative subgroup should still be managed with intensive risk factor control and guideline-directed lipid-lowering therapy, because their observed phenotype and event burden remain consistent with high lifetime ASCVD risk. Dietary counselling should accompany pharmacological treatment in both genotype-positive and genotype-negative patients with a clinically significant FH phenotype. Although diet alone is generally insufficient to normalize LDL-C in monogenic FH, a heart-healthy dietary pattern, avoidance of tobacco exposure, weight management, and regular physical activity remain important components of overall cardiovascular risk reduction.

The present study was not designed to test associations between pharmacogenetic variants and statin efficacy, LDL-C reduction, or statin-associated musculoskeletal symptoms. Historical sequencing results were generated on heterogeneous platforms and did not provide standardized star-allele/diplotype calls across the whole cohort. Furthermore, statin agent, dose, treatment duration, adherence, muscle symptoms, creatine kinase measurements, treatment discontinuation, and longitudinal LDL-C response were not collected prospectively in a harmonized manner. Therefore, PGx findings should be understood exclusively as observations relevant to future implementation, reporting, and the design of prospective studies. They cannot be interpreted as reliable patient-level predictors of statin response or intolerance.

This distinction is particularly important for statin therapy. Pharmacogenomic results can support statin selection and monitoring, especially when they concern SLCO1B1, ABCG2 or CYP2C9 and are interpreted according to validated guidance [8]. Evidence specifically obtained in FH populations remains limited; for example, an FH cohort study evaluated the relationship between SLCO1B1 rs4149056 and statin-associated muscle symptoms, illustrating the need for phenotype-specific and prospectively collected treatment data [27]. However, pharmacogenomics should not be used as a reason to withhold indicated lipid-lowering therapy in patients with FH or ACS [8,9,10,11,12]. In clinical practice, validated PGx guidance may support statin selection, dose adjustment, and monitoring when technically robust genotype-to-phenotype translation is available, while maintaining the urgency of LDL-C lowering [8,15,16].

Patients with ACS had lower measured TC and LDL-C concentrations than patients without ACS; however, these values were obtained during the acute event and may have been influenced by acute-phase changes and previous lipid-lowering treatment, rather than reflecting lower untreated lifetime LDL-C exposure [19,20]. If such values were entered into a multivariable model without context, the model could falsely suggest an inverse or protective relationship between LDL-C and ACS. The descriptive approach avoids this misleading interpretation. A small sample can still contribute to the literature when the research question is correctly framed [17,18]. Here, the useful outcome is not a new risk score but an implementation map: which results can be used diagnostically, which PGx genes require validated diplotype interpretation, which findings should remain non-actionable and what information must be collected prospectively for future precision-medicine studies. This is aligned with the practical goal of pharmacogenomics: moving from sequence data to safe, interpretable and clinically useful treatment decisions [8,15,16].

For future studies, a prospective design should collect baseline untreated LDL-C, post-treatment LDL-C response, statin type and dose, adherence, muscle symptoms, creatine kinase measurements when clinically indicated, drug discontinuation, non-statin therapy escalation and validated PGx diplotypes. Such data would allow pharmacogenomic hypotheses to be tested directly. Until then, small cohorts should be used to improve workflows, not to claim definitive gene-drug outcome associations.

### 4.1. Why No Regression Model Was Built

In this small, clinically selected, single-center descriptive cohort based on retrospectively analyzed records, we deliberately did not construct multivariable regression models. The limited number of patients with diagnostic FH-associated variants, heterogeneity in lipid-measurement timing (acute ACS values versus qualifying untreated LDL-C values), and absence of harmonized prospectively collected pharmacogenomic outcomes would have produced unstable and potentially misleading regression estimates. Instead, we used a descriptive implementation framework focused on diagnostic yield, clinical phenotype, and pharmacogenomic readiness, because this approach is more appropriate than multivariable risk modelling when sample size, heterogeneous measurement timing, and available outcome data limit reliable inference.

### 4.2. Limitations

This study has several limitations. It was a small, clinically selected, single-center descriptive cohort based on retrospectively analyzed clinical and genetic records; consequently, its findings may not be generalizable to other settings or populations. The number of patients carrying individual pathogenic variants was small. Lipid values in ACS patients were obtained during the acute phase and may not reflect the patient’s untreated baseline LDL-C exposure. Information on the duration, intensity, and adherence to lipid-lowering therapy before referral was not available in a fully standardized form for all patients. Consequently, observed lipid concentrations cannot be attributed solely to genetic background, particularly in patients with previous statin, ezetimibe, or other lipid-lowering treatment. Dietary history and adherence to dietary counseling were also not systematically captured. Although dietary measures alone are generally insufficient to normalize LDL-C in monogenic FH, a heart-healthy dietary pattern remains an important adjunct to pharmacological therapy and overall cardiovascular risk reduction. Sequencing platforms differed during the study period, and harmonized pharmacogenomic diplotype calling was not available across all patients. The study did not prospectively collect statin-associated musculoskeletal symptoms, adherence, dose changes or LDL-C response after therapy. Copy-number variant analysis was performed with different algorithms in the two subgroups (CNVkit for the targeted panel and the DRAGEN copy-number caller for whole-exome data), and copy-number calls were not confirmed by multiplex ligation-dependent probe amplification, so sensitivity for single-exon events may differ between workflows. Deep intronic and regulatory variants were within the analytical scope of the whole-exome design only for selected known positions and were not interrogated by the targeted panel. Polygenic risk scoring was not performed, so a polygenic contribution to hypercholesterolemia in the gene-negative subgroup could not be quantified. Reported variants were not confirmed by an orthogonal method such as Sanger sequencing, although all were called at high depth and passed the applied quality filters. Whole-exome samples analysed earlier in the study period were aligned to GRCh37/hg19 and later samples to GRCh38; variants are reported relative to fixed reference transcripts, but this heterogeneity should be noted. Pharmacogenetic loci were reported as individual variants rather than as star-allele diplotypes, and the CYP2D6 locus in particular cannot be reliably resolved by short-read sequencing. These limitations are central to the interpretation of this article and support its descriptive implementation framing.

## 5. Conclusions

In this small Lithuanian cohort of adults referred for suspected FH, pathogenic *LDLR* or *APOB* variants were identified in 27.1% of patients. The broader sequencing workflow demonstrates the practical overlap between diagnostic genomics and pharmacogenomics in precision lipidology. The most clinically useful approach is to separate monogenic FH diagnosis from pharmacogenomic prescribing support, avoid VUS-based clinical action and use validated statin PGx guidance only when technically robust genotype-to-phenotype interpretation is available. Small real-world cohorts can be valuable for identifying implementation barriers and reporting safeguards, but they should not be overinterpreted as definitive pharmacogenomic outcome studies. Importantly, the sizable proportion of gene-negative but clinically high-risk patients in this cohort emphasizes that FH care must continue to rely on careful phenotypic assessment and guideline-directed LDL-C lowering, irrespective of whether a monogenic variant is identified.

## Figures and Tables

**Table 1:** Clinical characteristics according to ACS status.

Variable	ACS Present, *n* = 28;	No ACS *n* = 20;	*p* Value
Age, years, mean (range)	50.2 (30–63)	44.6 (27–57)	0.033
Smoking, *n* (%)	9 (32.1)	2 (10.0)	0.160
BMI kg/m^2^, median (IQR)	29.4 (24.05–38.28)	26.57 (22.77–34.25)	0.053
Arterial hypertension, *n* (%)	26 (92.9)	6 (30)	<0.001
TC, mmol/L, median (IQR)	7.86 (6.05–10.46)	8.78 (6.22–14.91)	0.004
LDL-C, mmol/L, median (IQR)	5.5 (4.26–6.97)	6.65 (5.09–13)	0.001
HDL-C, mmol/L, median (IQR)	1.3 (0.84–1.94)	1.49 (0.64–2.04)	0.149
TG, mmol/L, median (IQR)	1.96 (0.5–7.84)	1.22 (0.35–4.2)	0.048
Diabetes mellitus, *n* (%)	2 (7.1)	1 (5.0)	1

Continuous variables are presented as mean (range) for age and as median (interquartile range [IQR]) for BMI and lipid variables. Categorical variables are presented as *n* (%). *p* values were calculated using the Mann–Whitney U test for continuous variables and Fisher’s exact test or the chi-square test for categorical variables, as appropriate. ACS—acute coronary syndrome; BMI—body mass index; TC—total cholesterol; LDL-C—low-density lipoprotein cholesterol; HDL-C—high-density lipoprotein cholesterol; TG—triglycerides.

**Table 2:** Patient-level diagnostic and non-diagnostic FH reporting categories in adults referred for clinically suspected familial hypercholesterolemia.

Genetic Reporting Category	Finding	Patients, *n* (%)	Clinical Interpretation
Diagnostic FH-associated findings	Pathogenic or likely pathogenic variants in established FH genes: *LDLR* c.1013G>A (p.Cys338Tyr), *LDLR* c.1829C>T (p.Ser610Phe), *LDLR* c.941-1_946del, and *APOB c*.10580G>A (p.Arg3527Gln)	13 (27.1)	Diagnostic result supporting monogenic FH; relevant for genetic counselling and cascade testing
VUS, common polymorphisms, or other non-diagnostic findings	Patients with VUS, common polymorphisms, exploratory variants, or no reportable variant; none had a pathogenic or likely pathogenic FH-associated variant identified within the analytical scope of the historical test used	35 (72.9)	Not diagnostic for monogenic FH; clinical management should remain based on phenotype, overall cardiovascular risk, and guideline-directed lipid-lowering therapy

*APOB*—apolipoprotein B, FH—familial hypercholesterolemia, *LDLR*—low-density lipoprotein receptor, *PCSK9*—proprotein convertase subtilisin/kexin type 9, VUS—variants of uncertain significance. Pharmacogenomic findings were interpreted separately as observations relevant to reporting and future implementation. They were not included as a mutually exclusive patient-level category because harmonized PGx star-allele/diplotype calling was not available across the historical sequencing platforms. The exact number of patients carrying at least one VUS could not be reconstructed reliably from the historical reports because VUS reporting and variant-level data availability differed between sequencing workflows.

**Table 3:** Pharmacogenomic interpretation framework for historical sequencing workflows used in the FH cohort.

Gene/Gene Group	Potential Role in Lipid-Lowering Therapy
SLCO1B1	Hepatic statin uptake; clinically relevant to statin exposure and SAMS risk
ABCG2	Efflux transporter; relevant particularly to rosuvastatin exposure
CYP2C9	Metabolism of selected statins, especially fluvastatin
CYP3A4/CYP3A5, HMGCR	Candidate or context-dependent relevance to statin exposure/response
ABCB1, SLCO1B3, SLCO2B1, CYP2D6, CYP2C19, UGT1A genes	Drug disposition or exploratory relevance depending on drug and variant
VUS in any gene	Uncertain clinical meaning

ABCG2—ATP-Binding Cassette subfamily G member 2, CYP2C9—Cytochrome P450 family 2 subfamily C member 9, CYP3A4/CYP3A5—Cytochrome P450 family 3 subfamily A members 4 and 5, CYP2D6—Cytochrome P450 family 2 subfamily D member 6, CYP2C19—Cytochrome P450 family 2 subfamily C member 19, HMGCR—3-Hydroxy-3-Methylglutaryl-CoA Reductase, SAMS—statin-associated musculoskeletal symptoms, SLCO1B3—Solute Carrier Organic Anion Transporter family member 1B3, SLCO2B1—Solute Carrier Organic Anion Transporter family member 2B1, UGT1A—UDP-Glucuronosyltransferase family 1 member A, VUS—variants of uncertain significance.

## Data Availability

The original contributions presented in this study are included in the article/Supplementary Materials. Further inquiries can be directed to the corresponding author.

## Supplementary Materials

The following supporting information can be downloaded at: https://www.mdpi.com/article/10.3390/medicina62101891/s1, Table S1: Historical sequencing workflows used in the familial hypercholesterolemia cohort and their analytical scope.

## Abbreviations

The following abbreviations are used in this manuscript:

ABCB1	ATP-Binding Cassette subfamily B member 1
ABCG2	ATP-Binding Cassette subfamily G member 2
ACS	acute coronary syndrome
APOB	apolipoprotein B
ASCVD	atherosclerotic cardiovascular disease
CD	cardiovascular disease
CYP2C9	Cytochrome P450 family 2 subfamily C member 9
CYP2C19	Cytochrome P450 family 2 subfamily C member 19
CYP2D6	Cytochrome P450 family 2 subfamily D member 6
CYP3A4/CYP3A5	Cytochrome P450 family 3 subfamily A members 4 and 5
DLCN	Dutch Lipid Clinic Network
ESC	European Society of Cardiology
FDB	Familial defective apolipoprotein B-100
FH	Familial hypercholesterolemia
HDL-C	high-density lipoprotein cholesterol
HMGCR	3-Hydroxy-3-Methylglutaryl-CoA Reductase
LAD	left anterior descending
LCX	left circumflex artery
LDL-C	low-density lipoprotein cholesterol
LDLR	low-density lipoprotein receptor
LDLRAP1	Low-Density Lipoprotein Receptor Adaptor Protein 1
PCSK9	proprotein convertase subtilisin/kexin type 9
PGx	pharmacogenomic
RCA	right coronary artery
SAMS	statin-associated musculoskeletal symptoms
SLCO1B1	Solute Carrier Organic Anion Transporter family member 1B1
SLCO1B3	Solute Carrier Organic Anion Transporter family member 1B3
SLCO2B1	Solute Carrier Organic Anion Transporter family member 2B1
TC	total cholesterol
TG	triglycerides
UGT1A	UDP-Glucuronosyltransferase family 1 member A
VUS	variants of uncertain significance
WES	whole-exome sequencing
