# Supplementary material for: Diagnostic Yield and Clinical Phenotype in a Lithuanian Familial Hypercholesterolemia Cohort with Heterogeneous Sequencing Platforms
Source: Medicina (Kaunas). 2026 Sep 29;62(10):1891. doi: 10.3390/medicina62101891 (PMC13706773; doi:10.3390/medicina62101891)
Supplement: Supplementary file 1 [file medicina-62-01891-s001.zip › medicina-4442836-supplementary.pdf]

Supplementary Table S1. Historical sequencing workflows used in the familial hypercholesterolemia cohort and their analytical scope.

Parameter	Targeted NGS panel	Whole-exome sequencing
Patients, n (%)	36 (75.0%)	12 (25.0%)
Testing laboratory	In-house laboratory	CeGaT GmbH, Tübingen, Germany
DNA extraction	Salting-out procedure	QIAsymphony DNA Kit on a QIAsymphony SP instrument
Library preparation and enrichment	Illumina DNA Prep with Enrichment and custom xGen Lockdown hybridisation capture panel	Twist Bioscience exome library preparation and enrichment kit
Sequencing platform	Illumina MiniSeq	Illumina NovaSeq
Read configuration	2 × 150 bp paired-end reads	2 × 100 bp paired-end reads
FH genes included in the analytical scope	LDLR, APOB, PCSK9, and LDLRAP1	Virtual panel containing LDLR, APOB, PCSK9, and LDLRAP1
Coverage information	All intended coding bases and canonical splice regions of the targeted panel were covered at ≥20×	Laboratory-reported mean on-target coverage: 150×; harmonized exon-level coverage metrics were not available retrospectively for all samples
Reference genome	GRCh38	GRCh37/hg19 or GRCh38, depending on the provider workflow at the time of analysis
Alignment and small-variant calling	BWA-MEM alignment; GATK Best Practices; GATK HaplotypeCaller	Illumina DRAGEN Bio-IT Platform
CNV analysis	CNVkit using a pooled reference from 30 independently sequenced samples	DRAGEN read-depth–based CNV caller
Variant interpretation	Franklin by Genoox; ACMG/AMP criteria; ClinGen FH VCEP specifications for LDLR, where applicable	Franklin by Genoox; ACMG/AMP criteria; ClinGen FH VCEP specifications for LDLR, where applicable

Important limitations	Deep intronic and regulatory variants were outside the targeted analytical scope; CNV calls were not orthogonally confirmed	Exon-level coverage and genome build were not fully harmonized across historical analyses; CNV calls were not orthogonally confirmed; deep intronic and regulatory variants were not systematically assessed
